# Baseline Physical Activity Behaviors and Relationships with Fitness in the Army Training at High Intensity Study

**DOI:** 10.3390/jfmk7010027

**Published:** 2022-03-08

**Authors:** Katie M. Heinrich, Aspen E. Streetman, Filip Kukić, Chunki Fong, Brittany S. Hollerbach, Blake D. Goodman, Christopher K. Haddock, Walker S. C. Poston

**Affiliations:** 1 Department of Kinesiology, Kansas State University, Manhattan, KS 66506, USA; aestreetman@ksu.edu (A.E.S.); bdgoodman@ksu.edu (B.D.G.); 2 Police Sports Education Center, Abu Dhabi Police, Abu Dhabi 253, United Arab Emirates; filip.kukic@gmail.com; 3 Institute for Implementation Science in Population Health, City University of New York Graduate School of Public Health & Health Policy, New York, NY 10027, USA; chunki.fong@sph.cuny.edu; 4 NDRI-USA, New York, NY 10001, USA; hollerbach@ndri-usa.org (B.S.H.); haddock@ndri-usa.org (C.K.H.); poston@ndri-usa.org (W.S.C.P.)

**Keywords:** exercise, army physical fitness test, army combat fitness test, officers, military, health, soldiers

## Abstract

United States Army soldiers must meet physical fitness test standards. Criticisms of the Army Physical Fitness Test (APFT) include limited testing of only aerobic and muscular endurance activity domains; yet, it is unclear what levels of aerobic and muscle strengthening activity may help predict performance in aspects of the new Army Combat Fitness Test (ACFT). This study explored relationships between baseline self-reported aerobic and muscle strengthening activities and APFT- and ACFT-related performance. Baseline participant data (N = 123) were from a cluster-randomized clinical trial that recruited active-duty military personnel (mean age 33.7 ± 5.7 years, 72.4% White, 87.0% college-educated, 81.5% Officers). An online survey was used for self-report of socio-demographic characteristics and weekly aerobic and muscle-strengthening physical activity behaviors. Participants also completed the APFT (2 min push-ups, 2 min sit-ups, 2-mile run) and ACFT-related measures (1-repetition maximum deadlift, pull-up repetitions or timed flexed arm hang, horizontal jump, and dummy drag). Bivariate logistic regression found greater aerobic and muscle-strengthening activity predicted better APFT performance, while better ACFT-related performance was predicted by greater muscle-strengthening activity. Although our data are mostly from mid-career officers, command policies should emphasize the new Holistic Health and Fitness initiative that encourages regular aerobic and muscle-strengthening physical activity for soldiers.

## 1. Introduction

High levels of physical activity benefit overall health and physical fitness levels [1,2,3]. Physical fitness is one of the most essential attributes of military personnel [4,5,6]. Indeed, the U.S. Army mandates that soldiers meet the physical demands of any combat or duty position throughout their career [6]. Given the occupational demand for physical readiness, there is sustained interest in understanding physical activity and its relationship to physical fitness among military personnel [6].

Meeting current physical activity guidelines requires regular aerobic and muscle-strengthening activities each week [3]. At least 150 min of moderate aerobic or 75 min of vigorous aerobic activity, or a combination of the two, along with two or more days of full-body muscle-strengthening per week is recommended [3]. For additional health and fitness benefits (e.g., improved aerobic capacity), double the amount of aerobic physical activity per week is recommended [3]. In the most recent Department of Defense (DoD) survey, 28.2% of military personnel (Army = 19.7%) did not meet aerobic physical activity recommendations, while 45.3% reported doing double the amount of aerobic physical activity per week (Army = 53.2%) [7]. For muscle-strengthening activity, 49.6% of military personnel (Army = 54.4%) reported 3+ days per week, with 25.2% reporting <1 day/week (Army = 18.7%), 25.2% (Army = 26.9%) reporting 1–2 days per week [7]. Across all service branches, junior enlisted personnel were the most physically active, while military officers were most likely to do <150 min/week of aerobic and 1–2 days/week of muscle-strengthening activities [7]. In addition, the two oldest age groups (i.e., 35–44 years, and 45 and older) were significantly less likely to report 3+ days per week of muscle-strengthening activity [7].

Physical activity helps develop and maintain physical fitness that supports soldier health, enhances performance on fitness assessments, and facilitates overall force readiness [5,8]. Previous research suggests poor physical fitness among soldiers can lead to inability to perform required job tasks, injury, or attrition [9]. Moreover, because not all military personnel have structured training or mandatory physical activity reporting requirements (e.g., National Guard, Reserves) [10,11], soldier physical activity may not be explicitly promoted or supported, leading to detrimental physical capacity and stress tolerance [5].

The U.S. Army has used a variety of fitness tests over time [6]. Although the earlier tests included obstacle courses and multiple fitness components, since 1980 the test was simplified to include push-ups, sit-ups, and a run [6]. The Army Physical Fitness Test (APFT) measures upper-body, abdominal, and cardiovascular endurance by max-effort attempts for 2 min of push-ups, sit-ups, and a 2-mile run. While the push-up event [12] and 2-mile run [13] are accepted field tests of muscular endurance and aerobic capacity, respectively, previous literature critiques the sit-up event as an inadequate assessment of abdominal endurance [14]. Additional criticism includes that the APFT measures are endurance events that do not simulate combat tasks such as dragging, lifting, carrying, and sprinting [15].

To address these concerns, the Army began developing the Army Combat Fitness Test (ACFT) in 2013 to mimic soldier tasks and enhance fitness in practical and beneficial ways related to combat situations [16]. The ACFT is part of the larger Holistic Health and Fitness (H2F) system implemented in 2020 by the Army to improve soldier health and fitness [16]. The ACFT officially replaced the APFT on 1 October 2020, although its roll-out as the official test is set for April 2022 due to delays from COVID-19 and challenges to gender-neutral scoring standards [17]. The ACFT includes a 3-repetition maximum (3RM) deadlift, standing power throw, hand-release push-up, sprint-drag-carry, leg tuck or low-plank hold, and a 2-mile run [18]. These fitness measures were chosen to better predict combat readiness for modern battlefield demands, and the shift from the APFT to the ACFT demonstrates a change in physical readiness training doctrine that focuses on full-body muscle-strengthening activities and cardiovascular endurance in parallel. To date, research has identified that functional motor competence [19] and body composition [20] are significantly related to ACFT performance, yet, comparisons between physical activity behaviors and the expanded ACFT fitness domains are lacking for military personnel.

This study aimed to report baseline physical fitness data of a varied cohort of military personnel, who were mostly mid-career officers attending graduate school at the Fort Leavenworth Command and General Staff College (CGSC). This was done by exploring how different levels of aerobic and muscle-strengthening activities were associated with performance on the APFT and on the ACFT-related measures. We hypothesized that soldiers’ performance of aerobic activities would predict APFT performance while muscle-strengthening activities would predict performance on ACFT-related measures.

## 2. Materials and Methods

### 2.1. Design and Participants

This study utilized baseline data from a 6-month cluster-randomized clinical trial conducted between 2014 and 2019 among active-duty US military personnel participating in a 6-month exercise intervention, the Army Training at High Intensity Study (ATHIS; https://clinicaltrials.gov/ct2/show/NCT02407093 accessed on 15 January 2022). Participants were recruited over four years primarily from the CGSC at Fort Leavenworth, KS, as well as some Reserves and National Guard personnel from the Manhattan, KS area. Inclusion criteria were having physical clearance to participate via the Physical Activity Readiness Questionnaire [21], willingness to adhere to the study protocol and complete assessments, and high likelihood of assignment to their post over the course of the study. Participants were excluded if they were on medical profile or had any medical conditions or injuries preventing exercise participation, were currently on administrative leave or assigned to administrative duties, were civilians or international military, or had an implanted electronic device or pacemaker. Female participants who were pregnant or lactating were also excluded from study participation.

Study recruitment followed a two-stage process where participants received initial information about the survey and were given the chance to ask questions from study investigators before completing a screening survey. Eligible participants were then contacted via email or phone to schedule initial study assessments. At their initial assessment visit participants completed written informed consent to enroll in the study. All study procedures were approved by Kansas State University’s Institutional Review Board (IRB; Approval #7162) and received administrative approval from the CGSC IRB.

### 2.2. Measures

#### 2.2.1. Survey

Physical-activity questions were from the 2011 DoD health-related behaviors survey [22]. Participants were asked to specify how often in the past 30 days they did moderate and vigorous aerobic and strength training (i.e., including bodyweight exercises or using weights or resistance training to increase muscle strength) activities by indicating the number of days per week and the average minutes per day. Demographic questions asked were also from the 2011 DoD survey (e.g., service branch, pay grade, gender, education) [22]. General health status and marital status were assessed using standardized questions from the Behavioral Risk Factors Surveillance System [23]. Participants were also asked to describe their current weight as “very underweight”, “slightly underweight”, “about the right weight”, “slightly overweight”, or “very overweight”. For analysis, very and slightly underweight were combined as “underweight” and slightly and very overweight were combined as “overweight”.

#### 2.2.2. Army Physical Fitness Test

This measure was used as it was the current standard at the time of study initiation. It was completed outdoors on a rubberized quarter-mile track. Procedures were followed exactly from the Army’s testing manual [24], except that participants were allowed to exceed the maximum number of repetitions. After warming up on their own, each participant completed 2 min of push-up repetitions to Army range of motion standards counted by trained study personnel. After at least 10 but less than 20 min of rest, each participant completed 2 min of sit-up repetitions to Army range of motion standards with their feet secured by another person and counted by trained study personnel. After a second 10 min minimum rest, participants completed the 2-mile run for time. Alternate aerobic events were not allowed.

#### 2.2.3. Strength, Power, and Muscular Endurance Measures

The following measures were chosen to assess domains of fitness important for military training that were not currently tested by the APFT and were all completed indoors. At the time of study development and initiation, the ACFT had not yet been developed. However, we were able to retrospectively identify how each of our study measures was related to an assessment included in the ACFT as identified in Table 1.

One Repetition Maximum (1RM) Deadlift. Hex bars (70 lb), rubberized and/or metal plates, and metal bar clamps were used for the deadlift. After receiving instructions on proper grip and form during the movement, participants warmed up by completing five repetitions at 40–60% of their 1RM, if known, or a relatively light weight if unknown. After a 1 min rest, participants completed three repetitions at 60–80% of 1RM. After another 1 min rest, participants had five attempts to establish a 1RM with 3–5 min of rest in-between attempts. The heaviest weight lifted was recorded.

Marine Corps Pull-up Test. This test was performed on a pull-up structure at a height where participants could hang from the bar without their feet touching the ground. Standardized procedures were used [25]. Participants were allowed to practice prior to initiating the test and could choose a pronated or supinated grip; however, they were not allowed to change their grip during the test. Participants then completed as many repetitions as possible by raising their body with their arms until their chin was above the bar and then lowering until their arms were fully extended. No whipping, kicking, or kipping of the body or legs or any leg movements were allowed. Both the grip and number of pull-ups completed were recorded. Participants unable to complete a single pull-up repetition were allowed to do a flexed-arm hang for time using standardized procedures [25]. Participants were allowed to jump or receive assistance to the starting position where their chin was above the bar and arms were flexed at the elbow. Time continued until the participant dropped off the bar or no longer maintained elbow flexion. Both the grip and total time were recorded.

Horizontal Jump. Participants were allowed to practice before completing three attempts at a standing horizontal jump for distance [26]. Participants had to “stick” the landing with both feet for the jump to be measured and the distance was measured from the starting line to the closest part of their heel. All successful jumps were recorded and the furthest was used for analysis.

Dummy Drag. Using procedures from the International Association of Fire Fighters Candidate Physical Ability Test [27], a 165 lb dummy was placed face up in the supine position with the head behind the start finish line. At the command “go”, each participant grasped the handles on a harness attached to the dummy’s upper torso and dragged it 35 ft backwards to and around a 270 lb stack of weights and then completely back across the starting line. The total time to complete the task was recorded.

### 2.3. Procedures

After completing informed consent at their first study visit, participants were emailed an individual link to an online survey via Qualtrics (Seattle, WA, USA). Fitness testing occurred on the second study visit. Preceding the testing, participants were instructed to drink plenty of fluids for 24 h, refrain from alcohol or caffeine for 6 h prior, refrain from eating or drinking for 3 h prior, and to wear appropriate clothing and footwear for physical exercise. Participants first reported to the outdoor track between 0500 and 0700 in groups based on preferred availability to complete the APFT. Next, they transitioned to an indoor facility to complete the rest of the fitness tests as described above. All testing was completed within 60–90 min depending on the size of the groups. All tests were administered and recorded by trained study staff members with either a military or fitness background.

### 2.4. Analysis

Data were entered into an excel database that was cleaned by a blinded statistician and then converted to a dataset readable by SAS software (Release 9.4 SAS Institute, Inc., Cary, NC, USA) for analysis. The self-reported aerobic physical activities were recoded as follows. The number of days per week in the past 30 days for moderate and vigorous activity were recoded from “about everyday”, “5–6 days per week”, “3–4 days per week”, “1–2 days per week”, “less than 1 day per week”, and “not at all in the past 30 days” to 7, 5.5, 3.5, 1.5, 0.7, and 0 days per week, respectively. The number of minutes of moderate and vigorous activity per day in the past 30 days were recoded from “60 or more minutes”, “30–59 min”, “20–29 min”, “less than 20 min” and “never in the past 30 days” to 60, 45, 25, 15, and 0 min per day, respectively. Then, the total minutes of aerobic physical activity data per week were calculated by multiplying the recoded days per week by number of estimated minutes per day and categorized as “not meeting physical-activity standards” (i.e., <150 min of moderate or <75 min of vigorous physical activity per week, or the combination of the two), “meeting physical-activity standards” (i.e., 150–299 min of moderate or 75–149 min of vigorous activity per week or their combination), or “exceeding physical-activity standards” (i.e., 300+ minutes of moderate or 150+ minutes of vigorous activity per week). Muscle-strengthening activity responses were categorized based on reported days per week as low (i.e., <1 day a week), medium (i.e., 1–2 days a week), and high (i.e., 3 or more days a week).

After computing frequencies for demographic characteristics, they were examined in relation to the weekly aerobic and the muscle-strengthening categories using ANOVA, and Chi-square or Fisher’s Exact test, as appropriate. Next, the three APFT events and five additional ACRT-related fitness tests were examined in relation to the weekly aerobic and the muscle-strengthening categories using ANOVA. Bivariate logistic regression analysis was used to examine the odds and effect size for each pair of aerobic physical activity and muscle-strengthening categories, in relation to performance on each fitness test. For those fitness tests with a statistically significant odds ratio, demographic characteristics that were associated with the activity standards were entered as covariates to determine the adjusted odds ratios. Finally, a visual representation of effect sizes was created for each pair of activity standards. Cohen’s d was the effect size measured, and statistical significance was set at *p* ≤ 0.05.

## 3. Results

### 3.1. Sample Characteristics

Baseline data were available for 123 participants. As shown in Table 2 participants were mostly middle-age for the Army (33.7 ± 5.7 years), male (76.4%), non-Hispanic White (72.4%) or non-Hispanic Black (10.6%). Most (87.0%) had at least a college degree, and almost half (45.5%) had a graduate degree. Over half (52.0%) reported very good to excellent health yet being overweight (58.5%).

Table 2 also displays these demographic characteristics for the participants overall and by aerobic physical activity and muscle-strengthening-activity frequency. Those who were younger (*p* = 0.022), less educated (*p* = 0.039), and who had better health (*p* = 0.007) and weight status (*p* = 0.029) were significantly more likely to meet the performance standard. Those who were not married were significantly more likely to report more days of muscle-strengthening activity (*p* = 0.024). These statistically significant social demographics were entered in the analysis as covariates.

### 3.2. Fitness Test Performance

Examination of fitness test performance by weekly aerobic activity and muscle-strengthening-frequency categories is shown in Table 3. Participants who reported greater weekly aerobic activity completed significantly more push-ups (0.004) and sit-ups (*p* = 0.002) and had faster 2-mile run times (*p* = 0.002) than those who were not meeting physical-activity standards. Participants who reported more days/week of muscle-strengthening activities did significantly more repetitions of push-ups (*p* = 0.001), sit-ups (*p* = 0.004), and pull-ups (*p* = 0.010), deadlifted more weight (*p* = 0.002), and jumped further (*p* = 0.044) than those reporting <1 day/week. Only flexed arm hang and dummy drag were similar between the muscle-strengthening-frequency groups, while all other performances were better in groups that reported higher frequencies of weekly muscle-strengthening activity.

### 3.3. Comparison by Physical Activity Categories

Categories of weekly aerobic activity and muscle-strengthening frequency are compared in Table 4. Bivariate logistic regression results showed that as compared to those not meeting physical-activity standards, those who met the aerobic standards had increased odds of completing additional sit-up repetitions (by 3%) and having a 1 min faster 2-mile run time (by 20%). After controlling for demographic covariates, only increased odds of having a 1 min faster 2-mile run time (by 43%) remained statistically significant. As compared to those not meeting aerobic physical-activity standards, those who exceeded them had increased odds of performing one more push-up repetition (by 5%), one sit-up repetition (by 6%), a 1 min faster 2-mile run time (by 32%), deadlifting an additional pound (by 1%), completing an additional pull-up repetition (by 11%), and a 1 s faster dummy drag time (by 8%). After demographic characteristics were added, those exceeding aerobic standards were still more likely than those who did not meet the standards to complete additional push-up repetitions (by 7%), sit-up repetitions (by 9%), and a 1 min faster 2-mile run time (by 55%). Comparison between those exceeding and meeting physical-activity standards only found higher odds of completing one more push-up repetition (by 3%), which was no longer significant after the demographic covariates were entered into the model.

Bivariate logistic regression results showed that those reporting medium versus low levels of muscle-strengthening activity had significantly greater odds of completing one more push-up repetition (by 3%), sit-up repetition (by 4%), and pull-up repetition (by 11%). These differences remained significant after including the demographic covariate (one additional push-up repetition = by 4%, sit-up repetition = by 4%, and pull-up repetition = by 13%). Those reporting high versus low levels of muscle-strengthening activity had significantly increase odds of completing one more push-up repetition (by 5%), sit-up repetition (by 6%), a 1 min faster 2-mile run time (by 18%), lifting 1 pound more deadlift weight (by 1%), one more pull-up repetition (by 17%), and jumping 1 cm further (by 2%). When adjusted for the demographic covariate, significant differences remained for push-ups (by 6%), sit-ups (by 6%), 2-mile run (by 27%), deadlift (by 1%), pull-ups (by 20%), and standing long jump (by 2%). Finally, comparison of those reporting high versus medium days of muscle-strengthening activity showed increased odds for lifting 1 pound more deadlift weight (by 1%), which remained significant after adding the demographic covariate (by 1%).

Table 5 visually summarizes the effect sizes for the fitness measures when compared between physical activity categories. The largest effect sizes were found for components of the APFT including between physical activity categories for high versus low categories of muscle-strengthening activity for push-ups and sit-up repetitions and for high versus low aerobic physical activity for sit-up repetitions and 2-mile run time. For ACFT-related measures, moderate effect sizes were found for greater levels of aerobic physical activity for pull-up repetitions, flexed arm hang time, and dummy drag time, while higher muscle strengthening activity had moderate effect sizes for deadlift weight, pull-up repetitions, and standing long jump distance.

## 4. Discussion

This study compared baseline fitness performance by self-reported levels of aerobic and muscle-strengthening activities for soldiers participating in an exercise intervention. Bivariate logistic regression results revealed that higher levels of aerobic and muscle-strengthening activity significantly predicted better APFT performance, while better performance on ACFT-related measures was significantly predicted by greater muscle-strengthening activity. Thus, the study hypothesis was partially supported. However, a separate examination of effect sizes found that greater levels of aerobic physical activity had moderate effects for performance of three ACFT-related tasks (i.e., pull-ups, flexed arm hang, dummy drag).

In comparison to recent DoD survey data, a greater percentage of our participants did not meet aerobic physical-activity standards, while fewer reported exceeding them [7]. For muscle-strengthening activity, a smaller percentage of our participants reported high muscle-strengthening frequency than found in the DoD survey [7]. This may be due to the fact that our study personnel were mostly mid-career officers, since the prior survey found greater aerobic and muscle-strengthening activity for junior enlisted personnel and younger age groups [7].

Study findings clearly reflect the positive relationship between physical activity and physical fitness performance for military personnel, as found by previous research [5,8]. Inconsistent training requirements can negatively affect soldier physical fitness capacity [5]. During our study period (i.e., 2015–2019), physical training requirements varied for CGSC students from no official requirements to 5 days per week of group physical training depending on the policies of the current Commander (email communication with David B. Batchelor, MS, COL-Retired, 10 July 2021). Moreover, the Commanding General can encourage soldiers to use time in the workday to prioritize exercise. Work commitments are a key barrier to physical activity reported by over 50% of military officers [28]. Physical fitness practices including maintaining consistent training requirements, emphasizing soldier physical fitness, and encouraging workday exercise, may encourage physical readiness. It is encouraging that the H2F initiative recognized the importance of moving beyond a one-size-fits-all training protocol to better serve individual soldiers and the Army as a whole. Recent research supports the notion that specific task-based training is essential to meet operational demands thus increasing soldier readiness [29].

Study measures were designed to assess more fitness domains than those measured by the APFT, similar to earlier physical fitness tests used in the Army [6]. Both push-ups and the 2-mile run have been deemed acceptable field tests of muscular endurance and aerobic capacity [12,13], and better performance on each were predicted by great aerobic and muscle-strengthening activity in this study. Addition of the deadlift, pull-up/flexed arm hang, long jump, and dummy drag allowed us to test more combat-specific tasks such as dragging and lifting [15]. While these measures are not identical to those in the current ACFT, they test similar fitness domains (e.g., strength = 1RM deadlift versus the 3RM of the ACFT; power = standing long jump versus standing power throw of the ACFT; muscular endurance = pull-up/flexed arm hang versus hand release push-up on the ACFT; casualty evacuation = dummy drag versus the sprint-drag-carry of the ACFT) [18].

Military professionals and researchers identify physical fitness and the physical demands of the operational environment as priority areas for filling gaps in military research [30]. As the Army adapts to the new requirements of 21st-century warfare, physical readiness remains a cornerstone characteristic of today’s soldiers. Shifting from the APFT to the ACFT is changing how Army soldiers train by focusing on full-body muscle-strengthening activities and cardiovascular endurance in parallel. This is perceived to better predict combat readiness despite lacking evidence regarding how ACFT performance compares with combat readiness. While our study did not assess combat readiness, we were able to show that increasing levels of self-reported physical activity, particularly muscle-strengthening activity, increased odds of better performance on measures closely related to the ACFT.

### 4.1. Strengths and Limitations

Our study had several strengths including successful recruitment over four years of active-duty military personnel enrolled in graduate school at CGSC or in the Reserves or National Guard that had varying fitness requirements. All fitness measures were conducted in person by trained study staff members at the same time of day in the morning. We did not examine ordering of fitness assessments in our study, and thus performance on the APFT may have affected performance on the ACFT-related measures. As well, we were unable to directly test the ACFT as it did not exist at the start of our study. While we used standardized DoD self-reported physical activity questions, we lacked objective assessment of actual physical activity behaviors. Previous research in non-military samples has found discrepancies between self-reported and objective measurements of physical activity [31]. Also, our sample size of 123 participants was small for comparisons between groups, which is why we reported effect sizes for our analyses. Results may not apply to younger enlisted soldiers or those at other Army installations.

### 4.2. Conclusions

Higher levels of both aerobic and muscle-strengthening activities significantly predicted better performance on the APFT, while higher muscle-strengthening activity was predictive of performance on ACFT-related measures. As the Army continues to implement the H2F program, continuing emphasis on increasing both domains of physical activity is important and may particularly benefit military officers enrolled in graduate degree programs at CGSC.

## Figures and Tables

**Table 1:** Comparison of Army Training at High Intensity Study (ATHIS) measures to strength, power, and muscular endurance measures included in the Army Combat Fitness Test (ACFT).

Fitness Domain	Physical Measure	ATHIS Measure	ACFT Measure
Muscular strength	Maximal force	One-repetition maximum deadlift	Three-repetition maximum deadlift
Power	Jumps and throws	Standing long jump	Standing power throw
Upper-body muscular endurance	Short-term sustained force or average power	Marine Corps pull-up test/flexed arm hang	Hand-release push-ups
Full-body muscular endurance	Short-term sustained force or average power	Dummy drag	Sprint-drag-carry

**Table 2:** Participant demographic characteristics by physical activity categories.

	Overall	Weekly Aerobic Activity				Muscle-Strengthening Frequency			
		Not Meeting Physical-Activity Standards ^a^	Meeting Physical-Activity Standards ^b^	Exceeding Physical-Activity Standards ^c^	*p*-Value	<1 Day/wk	1–2 Days/wk	3+ Days/wk	*p*-Value
	123 (100%)	36 (29.3%)	47 (38.2%)	40 (32.5%)		31 (25.2%)	43 (35.0%)	49 (39.8%)	
Age (Mean/St. Dev.)	33.4 (5.7)	35.5 (3.9)	33.4 (6.2)	31.7 (5.9)	0.022	35.2 (5.7)	33.5 (4.4)	32.2 (6.4)	0.057
Gender					0.258				0.692
Male	94 (76.4%)	25 (69.4%)	35 (74.5%)	34 (85.0%)		24 (77.4%)	31 (72.1%)	39 (79.6%)	
Female	29 (23.6%)	11 (30.6%)	12 (25.5%)	6 (15.0%)		7 (22.6%)	12 (27.9%)	10 (20.4%)	
Race/Ethnicity					0.233				0.089
Hispanic	6 (4.9%)	1 (2.8%)	2 (4.3%)	3 (7.5%)		1 (3.2%)	6 (11.6%)	0 (0.0%)	
Non-Hispanic White	89 (72.4%)	22 (61.1%)	37 (78.7%)	30 (75.0%)		20 (64.5%)	30 (69.8%)	39 (79.6%)	
Non-Hispanic Black	13 (10.6%)	4 (11.1%)	5 (10.6%)	4 (10.0%)		6 (19.4%)	2 (4.7%)	5 (10.2%)	
Other	15 (12.2%)	9 (25.0%)	3 (6.4%)	3 (7.5%)		4 (12.9%)	6 (14.0%)	5 (10.2%)	
Annual household income					0.228				0.459
<= USD 75,000	34 (27.6%)	6 (16.7%)	16 (34.0%)	12 (30.0%)		7 (22.6%)	9 (20.9%)	18 (36.7%)	
USD 75,001–100,000	47 (38.2%)	13 (36.1%)	21 (44.7%)	13 (32.5%)		12 (38.7%)	19 (44.2%)	16 (32.7%)	
>USD 100,000	42 (34.1%)	17 (47.2%)	10 (21.3%)	15 (37.5%)		12 (38.7%)	15 (34.9%)	15 (30.6%)	
Highest level of education					0.039				0.139
No college degree	16 (13.0%)	0 (0.0%)	7 (14.9%)	9 (22.5%)		3 (9.7%)	2 (4.7%)	11 (22.4%)	
Undergraduate degree	27 (22.0%)	8 (22.2%)	9 (19.1%)	10 (25.0%)		4 (12.9%)	11 (25.6%)	12 (24.5%)	
Some graduate degree	24 (19.5%)	11 (30.6%)	8 (17.0%)	5 (12.5%)		7 (22.6%)	8 (18.6%)	9 (18.4%)	
Graduate degree	56 (45.5%)	17 (47.2%)	23 (48.9%)	16 (40.0%)		17 (54.8%)	22 (51.2%)	17 (34.7%)	
Married					0.542				0.024
Yes	78 (63.9%)	25 (69.4%)	30 (65.2%)	23 (57.5%)		25 (80.6%)	28 (66.7%)	25 (51.0%)	
No	44 (34.1%)	11 (30.6%)	16 (34.8%)	17 (42.5%)		6 (19.4%)	14 (33.3%)	24 (49.0%)	
General health status					0.007				0.071
Excellent	16 (13.0%)	1 (2.8%)	5 (10.6%)	10 (25.0%)		3 (9.7%)	3 (7.0%)	10 (20.4%)	
Very good	48 (39.0%)	11 (30.6%)	17 (36.2%)	20 (50.0%)		9 (29.0%)	17 (39.5%)	22 (44.9%)	
Good	52 (42.3%)	21 (58.3%)	22 (46.8%)	9 (22.5%)		16 (51.6%)	19 (44.2%)	17 (34.7%)	
Fair	7 (5.7%)	3 (8.3%)	3 (6.4%)	1 (2.5%)		3 (9.7%)	4 (9.3%)	0 (0.0%)	
Current weight status					0.029				0.165
Underweight	7 (5.7%)	1 (2.8%)	1 (2.1%)	5 (12.5%)		1 (3.2%)	3 (7.0%)	3 (6.1%)	
Right weight	44 (35.8%)	12 (33.3%)	13 (27.7%)	19 (47.5%)		6 (19.4%)	17 (39.5%)	21 (42.9%)	
Overweight	72 (58.5%)	23 (63.9%)	33 (70.2%)	16 (40.0%)		24 (77.4%)	23 (53.5%)	25 (51.0%)	
Rank					0.792				0.081
Enlisted	23 (18.7%)	4 (11.1%)	9 (19.1%)	10 (25.0%)		5 (16.1%)	4 (9.3%)	14 (28.6%)	
Officers	100 (81.3%)	32 (88.9%)	38 (80.9%)	30 (75.0%)		26 (83.9%)	39 (90.7%)	35 (71.4%)	

^a^ <150 min of moderate or <75 min of vigorous activity per week; ^b^ 150–299 min of moderate or 75–149 min of vigorous activity per week, ^c^ 300+ minutes of moderate or 150+ min of vigorous activity per week.

**Table 3:** Comparison of fitness test performance by physical activity categories.

Weekly Aerobic Activity							
Activity	Not Meeting Physical-Activity Standards		Meeting Physical-Activity Standards		Exceeding Physical-Activity Standards		
	Mean	St. Dev.	Mean	St. Dev.	Mean	St. Dev.	*p*-Value
Push-ups (reps)	45.3	16.3	48.4	19.4	58.4	17.2	0.004
Sit-ups (reps)	58.1	14.3	64.5	13.9	69.9	14.7	0.002
2-mile run (min.s)	17.5	2.2	16.4	2.3	15.5	2.3	0.002
Deadlift (pounds)	268.7	70.0	288.3	89.3	303.4	72.4	0.167
Pull-ups (reps)	4.8	4.4	5.8	5.0	7.7	6.1	0.052
Flexed arm hang (s)	16.4	14.4	17.8	12.2	26.7	21.7	0.418
Standing long jump (cm)	188.3	34.9	191.4	35.5	194.4	45.4	0.792
Dummy drag (s)	25.5	15.7	23.7	12.2	19.7	5.6	0.089
**Muscle-Strengthening Frequency**							
**Activity**	**<1 day/week**		**1–2 days/week**		**3+ days/week**		
	**Mean**	**St. Dev.**	**Mean**	**St. Dev.**	**Mean**	**St. Dev.**	***p*** **-Value**
Push-ups (reps)	41.9	15.6	49.8	16.1	57.5	19.9	0.001
Sit-ups (reps)	57.0	13.5	65.6	15.0	68.2	14.2	0.004
2-mile run (min.s)	17.2	2.2	16.2	2.3	16.1	2.5	0.099
Deadlift (pounds)	263.1	68.0	271.1	58.4	317.9	92.2	0.002
Pull-ups (reps)	3.8	3.8	6.2	5.5	7.5	5.6	0.010
Flexed arm hang (s)	20.1	11.9	17.6	14.5	20.1	20.1	0.933
Standing long jump (cm)	41.9	15.6	49.8	16.1	57.5	19.9	0.044
Dummy drag (s)	57.0	13.5	65.6	15	68.2	14.2	0.126

**Table 4:** Bivariate logistic regression results comparing differences between levels of physical activity and fitness test performance.

Weekly Aerobic Activity ^1^															
Activity	Meeting Physical-Activity Standards Compared to Not Meeting Standards					Exceeding Physical-Activity Standards Compared to Not Meeting Standards					Exceeding Physical-Activity Standards Compared to Meeting Standards				
	Effect Size	Unadj. OR (95% CI)	*p*-Value	Adj.OR (95% CI)	*p*-Value	Effect Size	Unadj. OR (95% CI)	*p*-Value	Adj. OR (95% CI)	*p*-Value	Effect Size	Unadj. OR (95% CI)	*p*-Value	Adj. OR (95% CI)	*p*-Value
Push-ups (reps)	0.17	1.01 (0.99–1.04)	0.367	-	-	0.78	1.05 (1.02–1.08)	0.003	1.07 (1.02–1.13)	0.010	0.55	1.03 (1.01–1.06)	0.017	1.02 (0.99–1.05)	0.140
Sit-ups (reps)	0.45	1.03 (1.00–1.07)	0.049	1.04 (1.00–1.09)	0.056	0.81	1.06 (1.02–1.10)	0.002	1.09 (1.02–1.16)	0.009	0.38	1.03 (1.00–1.06)	0.090	-	-
2-mile run (min.s)	0.49	0.80 (0.65–0.99)	0.039	0.57 (0.40–0.81)	0.002	0.89	0.68 (0.54–0.86)	0.001	0.45 (0.27–0.76)	0.003	0.39	0.85 (0.69–1.03)	0.094	-	-
Deadlift (pounds)	0.25	1.03 (1.00–1.01)	0.287	-	-	0.49	1.01 (1.00–1.01)	0.044	1.01 (1.00–1.02)	0.059	0.19	1.00 (1.00–1.01)	0.392	-	-
Pull-ups (reps)	0.21	1.05 (0.95–1.15)	0.337	-	-	0.55	1.11 (1.01–1.21)	0.026	1.12 (0.96–1.31)	0.137	0.34	1.06 (0.98–1.15)	0.118	-	-
Flexed arm hang (s)	0.11	1.01 (0.94–1.08)	0.793	-	-	0.57	1.04 (0.97–1.11)	0.288	-	-	0.53	1.04 (0.97–1.11)	0.262	-	-
Standing long jump (cm)	0.09	1.00 (0.99–1.02)	0.687	-	-	0.15	1.00 (0.99–1.02)	0.514	-	-	0.07	1.00 (0.99–1.01)	0.731	-	-
Dummy drag (s)	0.13	0.99 (0.96–1.02)	0.573	-	-	0.54	0.92 (0.86–0.99)	0.036	0.93 (0.83–1.04)	0.184	0.45	0.95 (0.89–1.01)	0.075	-	-
**Muscle-Strengthening Frequency ^2^**															
**Activity**	**1–2 days/week compared to** **<1 day/week**					**3+ days/week compared to** **<1 day/week**					**3+ days/week compared to** **1–2 days/week**				
	**Effect size**	**Unadj. OR (95% CI)**	***p*** **-value**	**Adj. OR (95% CI)**	***p*** **-value**	**Effect size**	**Unadj. OR (95% CI)**	***p*** **-value**	**Adj. OR (95% CI)**	***p*** **-value**	**Effect size**	**Unadj. OR (95% CI)**	***p*** **-value**	**Adj. OR (95% CI)**	***p*** **-value**
Push-ups (reps)	0.50	1.03 (1.00–1.06)	0.043	1.04 (1.01–1.08)	0.013	0.88	1.05 (1.02–1.08)	0.001	1.06 (1.03–1.09)	<.001	0.43	1.02 (1.00–1.05)	0.054	-	-
Sit-ups (reps)	0.60	1.04 (1.01–1.08)	0.018	1.04 (1.01–1.08)	0.021	0.81	1.06 (1.02–1.10)	0.002	1.06 (1.02–1.10)	0.004	0.18	1.01 (0.98–1.04)	0.395	-	-
2-mile run (min.s)	0.44	0.82 (0.66–1.02)	0.076	-	-	0.47	0.82 (0.67–1.00)	0.050	0.73 (0.58–0.93)	0.009	0.04	0.98 (0.82–1.17)	0.792	-	-
Deadlift (pounds)	0.13	1.00 (1.00–1.01)	0.590	-	-	0.68	1.01 (1.00–1.01)	0.009	1.01 (1.00–1.02)	0.004	0.62	1.01 (1.00–1.01)	0.009	1.01 (1.00–1.01)	0.007
Pull-ups (reps)	0.52	1.11 (1.00–1.23)	0.045	1.13 (1.01–1.26)	0.026	0.79	1.17 (1.05–1.30)	0.004	1.20 (1.07–1.36)	0.003	0.23	1.04 (0.97–1.12)	0.277	-	-
Flexed arm hang (s)	0.19	0.98 (0.91–1.06)	0.676	-	-	0.00	1.00 (0.94–1.06)	0.999	-	-	0.14	1.01 (0.95–1.07)	0.760	-	-
Standing long jump (cm)	0.20	1.01 (0.99–1.02)	0.409	-	-	0.62	1.02 (1.00–1.03)	0.013	1.02 (1.01–1.04)	0.007	0.34	1.01 (1.00–1.02)	0.117	-	-
Dummy drag (s)	0.13	1.01 (0.97–1.05)	0.602	-	-	0.38	0.96 (0.91–1.01)	0.111	-	-	0.42	0.96 (0.91–1.01)	0.087	-	-

^1^ Covariates: age, highest level of education, general health status, current weight status; ^2^ Covariate: married.

**Table 5:** Visual comparison of effect sizes by physical activity category for each fitness measure.

Activity	Low ^a^ versus Medium ^b^ Aerobic Activity	Low ^d^ Versus Medium ^e^ Muscle-Strengthening	Low versus High ^c^ Aerobic Activity	Low versus High ^f^ Muscle-Strengthening	Medium versus High Aerobic Activity	Medium versus High Muscle-Strengthening
Push-ups (reps)	Trivial	Moderate	Moderate	Large	Moderate	Small
Sit-ups (reps)	Small	Moderate	Large	Large	Small	Trivial
2-mile run (min.s)	Small	Small	Large	Small	Small	Trivial
Deadlift (pounds)	Small	Small	Small	Moderate	Trivial	Moderate
Pull-ups (reps)	Small	Moderate	Moderate	Moderate	Small	Small
Flexed arm hang (s)	Trivial	Trivial	Moderate	Trivial	Moderate	Trivial
Standing long jump (cm)	Trivial	Small	Trivial	Moderate	Trivial	Small
Dummy drag (s)	Trivial	Trivial	Moderate	Small	Small	Small

Trivial < 0.20; Small = 0.2–0.5; Medium = 0.5–0.8; Large = 0.8–1.3; Very large > 1.3; ^a^ Low = not meeting aerobic physical-activity standards; ^b^ Medium = meeting aerobic physical-activity standards; ^c^ High = exceeding aerobic physical-activity standards; ^d^ Low = <1 day/week; ^e^ 1–2 days/week; ^f^ 3+ days/week.

## Data Availability

Data are available by request from the corresponding author.
